# Generation of *Trichoderma atroviride* mutants with constitutively activated G protein signaling by using geneticin resistance as selection marker

**DOI:** 10.1186/1756-0500-5-641

**Published:** 2012-11-17

**Authors:** Sabine Gruber, Markus Omann, Carolina Escobar Rodrìguez, Theresa Radebner, Susanne Zeilinger

**Affiliations:** 1 Research Area Molecular Biotechnology and Microbiology, Institute of Chemical Engineering, Vienna University of Technology, Gumpendorferstrasse 1a, Wien, Austria; 2 Current address: Zuckerforschung Tulln GmbH, Josef-Reiter-Strasse 21-23, Tulln, Austria

**Keywords:** Fungi, Trichoderma, Genetic transformation, Geneticin, G protein signaling, Mycoparasitism

## Abstract

**Background:**

Species of the fungal genus *Trichoderma* are important industrial producers of cellulases and hemicellulases, but also widely used as biocontrol agents (BCAs) in agriculture. In the latter function *Trichoderma* species stimulate plant growth, induce plant defense and directly antagonize plant pathogenic fungi through their mycoparasitic capabilities. The recent release of the genome sequences of four mycoparasitic *Trichoderma* species now forms the basis for large-scale genetic manipulations of these important BCAs. Thus far, only a limited number of dominant selection markers, including Hygromycin B resistance (*hph*) and the acetamidase-encoding *amdS* gene, have been available for transformation of *Trichoderma* spp. For more extensive functional genomics studies the utilization of additional dominant markers will be essential.

**Results:**

We established the *Escherichia coli* neomycin phosphotransferase II-encoding *nptII* gene as a novel selectable marker for the transformation of *Trichoderma atroviride* conferring geneticin resistance. The *nptII* marker cassette was stably integrated into the fungal genome and transformants exhibited unaltered phenotypes compared to the wild-type. Co-transformation of *T. atroviride* with *nptII* and a constitutively activated version of the Gα subunit-encoding *tga3* gene (*tga3*^*Q207L*^) resulted in a high number of mitotically stable, geneticin-resistant transformants. Further analyses revealed a co-transformation frequency of 68% with 15 transformants having additionally integrated *tga3*^*Q207L*^ into their genome. Constitutive activation of the Tga3-mediated signaling pathway resulted in increased vegetative growth and an enhanced ability to antagonize plant pathogenic host fungi.

**Conclusion:**

The neomycin phosphotransferase II-encoding *nptII* gene from *Escherichia coli* proved to be a valuable tool for conferring geneticin resistance to the filamentous fungus *T. atroviride* thereby contributing to an enhanced genetic tractability of these important BCAs.

## Background

*Trichoderma* species are among the most frequently isolated soil fungi. While *Trichoderma reesei* is an efficient producer of cellulolytic and hemicellulolytic enzymes for industrial applications, other species such as the mycoparasites *Trichoderma atroviride, Trichoderma harzianum,* and *Trichoderma virens* represent important biocontrol agents (BCAs) applied in agriculture [1]. *Trichoderma-*based BCAs rely on the capability of these fungi to establish themselves in the plant rhizosphere, to stimulate plant growth and to induce plant defense against pathogens in addition to their ability to antagonize plant-pathogenic fungi by mycoparasitism [1-3]. Based on the fact that more than 60% of all registered biofungicides used for plant disease control are based on *Trichoderma*[4], there is an increasing interest to understand the modes of action of these fungi and the underlying molecular processes in greater detail. To gain a deeper understanding on how mycoparasitism in *Trichoderma* is induced by living host fungi intracellular signal transduction pathways have been investigated. Studies with both *T. atroviride* and *T. virens* revealed important roles of G protein-coupled receptors and heterotrimeric G proteins [5-10]. In *T. atroviride*, the Tga3 Gα protein was shown to regulate mycoparasitism-relevant processes, such as the attachment to the host fungus, and the production of cell wall-degrading enzymes and antifungal secondary metabolites. According to these essential functions, *tga3* gene deletion mutants were avirulent, i.e. unable to attack and lyse host fungi [10].

For the genetic manipulation of *T. atroviride* various transformation techniques, inlcuding biolistic, *Agrobacterium*-mediated, and protoplast-based methods have been established [11]. These approaches basically rely on only two dominant selection markers, the Hygromycin B resistance-conferring *hph* gene and the acetamidase-encoding *amdS* gene enabling the fungus to grow on acetamide as sole nitrogen source [12]. While *hph* proved to be a reliable marker for gene deletion as well as the ectopic integration of expression and silencing constructs in *T. atroviride*, transformation with *amdS* may result in background growth especially when the integrating DNA rendered transformants less viable. In order to facilitate serial gene deletions and re-transformations in this important biocontrol fungus, the establishment of additional selection markers is crucial.

The neomycin phosphotransferase II-encoding *nptII* gene probably is the most widely used selectable marker for the transformation of plants [13]. In recent years, *nptII* has been applied for the transformation of filamentous fungi revealing its usefulness in these organisms [14-18].

Here we report the establishment of the *nptII* gene as a dominant selectable marker for the genetic transformation of *T. atroviride* using geneticin (G418) resistance. Subsequently, *nptII* was applied in co-transformation experiments to generate mutants expressing a constitutively active version of the *tga3* Gα subunit-encoding gene. The consequences of the constitutive activation of Tga3-mediated signaling on *T. atroviride* growth, conidiation and mycoparasitism are presented.

## Results and discussion

### Sensitivity of untransformed *T. atroviride* to geneticin

**Figure 1:**
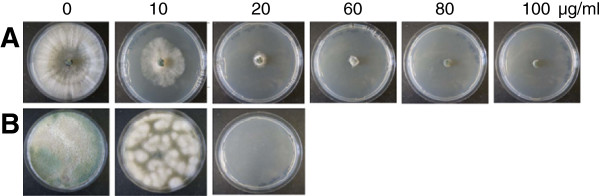
**Sensitivity of untransformed *T. atroviride *mycelia and conidia to geneticin (G418).** The sensitivity of *T. atroviride* P1 to geneticin was assessed by placing a mycelial agar plug (**A**) or 10^3^ fungal conidia (**B**) on PDA plates containing increasing concentrations of the antibiotic.

The sensitivity of *T. atroviride* strain P1 to geneticin was tested prior to transformation. Fungal growth from mycelial agar plug inocula placed on PDA plates supplemented with increasing amounts of geneticin were effectively impaired by concentrations of 10–60 μg/ml. Mycelia growth was completely inhibited by concentrations exceeding 60 μg/ml (Figure 1A). Conidial germination was completely blocked by concentrations of ≥ 20 μg/ml (Figure 1B). Based on these results, a geneticin concentration of 80 μg/ml was used for the selection of transformants.

### Expression of the *nptII* gene confers geneticin resistance to *T. atroviride*

**Figure 2:**
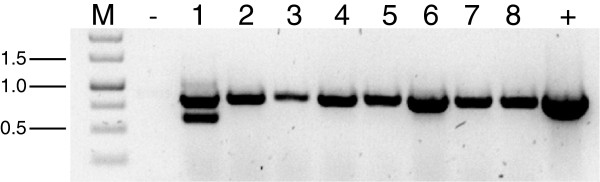
**Screening of *T. atroviridec *transformants for the presence of the *nptII *gene.** Genomic DNA was isolated from geneticin-resistant transformants and screened by PCR for the presence of the *nptII* gene using primers pII99Fw and pII99Rev (Table 1) which yielded a specific amplicon of 888-bp. The ~600-bp band amplified from transformant 1 indicates the presence of an additional incomplete *nptII* gene copy. M, DNA size standard; -, wild-type; 1–8, transformants; +, plasmid pII99.

**Figure 3:**
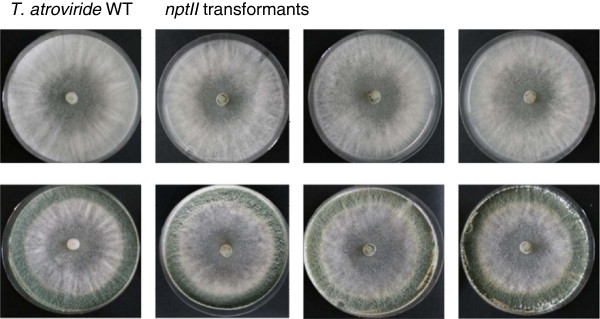
**Phenotype of geneticin-resistant transformants. ***T. atroviride* wild-type and three geneticin-resistant transformants harboring the *nptII* gene were grown on PDA at 28°C with short daily exposure to daylight. Pictures were taken after 5 days (upper row) and 10 days (lower row).

*T. atroviride* protoplasts were transformed with 10 μg of the circular plasmid pII99 [19]. Within 3–4 days, colonies of various sizes emerged on selective medium (PDA supplemented with 80 μg/ml geneticin). 14 colonies were isolated and transferred individually on a fresh set of selection plates, on which 8 colonies survived. After purification of these geneticin-resistant transformants to mitotic stability by three rounds of single spore isolation, integration of the *nptII* gene was examined. PCR analysis with oligonucleotides pII99Fw and pII99Rev (Table 1) resulted in the expected 888-bp amplicon in all tested transformants (Figure 2). To confirm that the *nptII* gene product itself has no unwanted phenotypic effects, fungal growth rates, conidiation behavior and mycoparasitic abilities of the transformants were assessed in comparison to the wild-type. All tested transformants exhibited an unaltered phenotype (Table 2, Figure 3), demonstrating that the *nptII* gene is a suitable selectable marker for transformation of *T. atroviride*.

**Table 1:** Oligonucleotides used

**name**	**forward primer (5**^**′**^** to 3**^**′**^**)**	**reverse primer (5**^**′**^** to 3**^**′**^**)**
*tga3Phusion*	ACGTTGGCGGGCTAAGGAGTGAACG	CAAACATGCTAAAGAAGAACGATC
*pII99*	ACTCGTCAAGAAGGCGATAGAAGGC	CAAGCAAGGTAAGTGAACGACCCG
*tga3Probe*	GAATCCAAACCTCCTCGTCC	GCAGAATCCATCAGGTAAAACTC
*tga3PromFw*	CATCAATCCATCATCAGTCCAAG	
*tga3TermRev*		CCTCTCTCATTTGCTCAACTC
*M13Fw*	GTTTTCCCAGTCACGAC	
*tga3LocusCheck*	CTTGGAGTCTGCGTGCG	
*Mut-tga3*	GACTACCAAGCAGAATCCGG	TGATTGAACCGCCATAGCAG

**Table 2:** **Phenotypic characteristics of *nptII *transformants and *nptII + tga3 ***^***Q207L ***^**co-transformants**

**Strain**	**Growth rate (mm/10 h)**	**Number of conidia (x10**^**9**^**)**
**Wild-type**	0.51 ± 0.03 (n=6)	7.4 ± 1.3 (n=3)
***nptII*only**	0.52 ± 0.02 (n=6)	11.1 ± 2.2 (n=3)
***tga3***^***Q207L***^***3/3***	0.63 ± 0.02 (n=6)	10.0 ± 3.4 (n=3)
***tga3***^***Q207L***^***4/5***	0.63 ± 0.02 (n=6)	7.1 ± 2.1 (n=3)
***tga3***^***Q207L***^***2/1***	0.62 ± 0.03 (n=6)	8.3 ± 1.7 (n=3)

### Co-transformation of *T. atroviride* with *nptII* and *tga3*^Q207L^

The *tga3* gene encodes the class III adenylate cyclase-activating Gα subunit of *T. atroviride*[10]. To generate a constitutively activated version of Tga3, a point mutation resulting in the replacement of glutamine by leucine at position 207 of the gene *(tga3*^*Q207L*^) was introduced. This single amino acid exchange has previously been shown to result in an impairment of the intrinsic GTPase activity of the corresponding Gα subunits [20], leading to a constitutively active protein which stimulates downstream effectors even in the absence of receptor activation.

**Figure 4:**
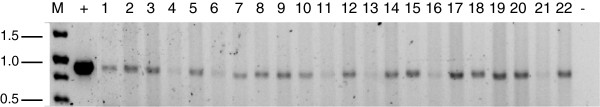
**Screening of *T. atroviride *co-transformants for the presence of the *nptII *gene.** Genomic DNA was isolated from 22 geneticin-resistant transformants resulting from co-transformation of *T. atroviride* P1 with plasmids pKAtga3 and pII99 and analyzed by PCR for integration of the *nptII* gene using primers pII99Fw and pII99Rev. M, DNA size standard; +, plasmid pII99; 1–22, transformants; -, wild-type.

Mutants expressing the constitutively active *tga3*^Q207L^ version were generated by co-transforming *T. atroviride* protoplasts with plasmids pKAtga3 (harboring *tga3*^Q207L^) and pII99. 30 colonies were isolated and transferred individually on PDA-geneticin selection plates of which 22 colonies developed. After purifying these transformants to mitotic stability, integration of the *nptII* gene into their genomes was examined by PCR as described above. Again, the expected 888-bp fragment could be amplified from all 22 transformants (Figure 4), proving the suitabilty of this selection marker for *T. atroviride* once more.

### Screening for *tga3*^Q207L^ in geneticin-resistant transformants

**Figure 5:**
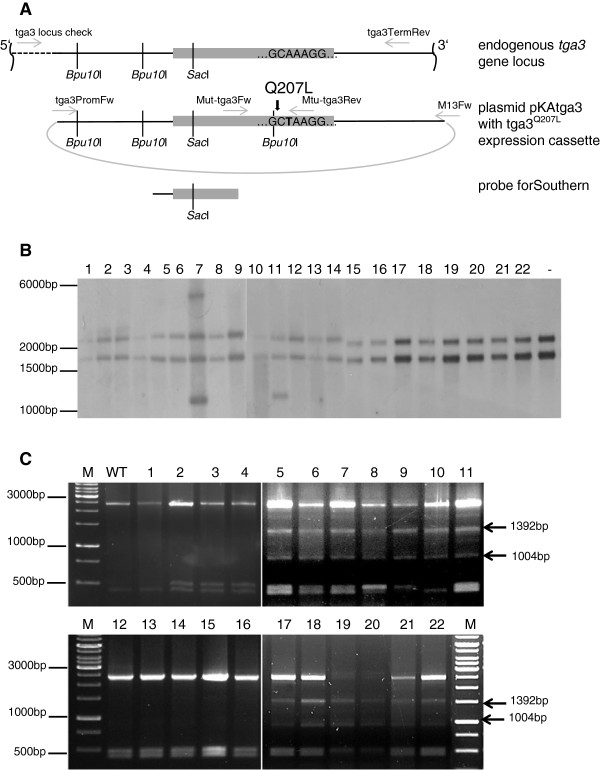
**Analysis of *T. atroviride *co-transformants for the presence of the *tga3***^***Q207L ***^**gene.** (**A**) Schematic drawing of the endogenous *tga3* gene locus and of the transforming plasmid pKAtga3 harboring the plasmid pKAtga3 with *tga3*^*Q207L*^expression cassette gene including 1030-bp of the 5^′^ and 783-bp of the 3^′^ non-coding regions. The point mutation resulting in the Q207L amino acid exchange is indicated by a bold type arrow. Grey arrows indicate primers used for the PCR-based approaches. (**B**) Southern blot of genomic DNA digested with *Sac*I and hybridized with a probe containing ~ 1118-bp of the *tga3* gene. The two bands of 1703-bp and 2272-bp present in all lanes including the wild-type (−) correspond to the endogenous *tga3* gene copy. The presence of additional bands in co-transformants 3/3 (lane 7) and 4/5 (lane 11) indicate ectopic integration of the *tga3*^*Q207L*^ gene. (**C**) The *tga3* gene locus was amplified by PCR as given in the Methods section and the resulting 3377-bp amplicon digested with *Bpu10*I. As an additional *Bpu10*I recognition site was generated in *tga3*^*Q207L*^ by introducing the A to T mutation, *Bpu10*I digestion resulted in the two indicative fragments (1004-bp, 1392-bp) in co-transformants with homologous integration of *tga3*^*Q207L*^ whereas the 2396-bp fragment remained undigested in the wild-type and ectopic co-transformants 3/3 (lane 12) and 4/5 (lane 13). WT; wildtype; M, DNA ladder.

**Figure 6:**
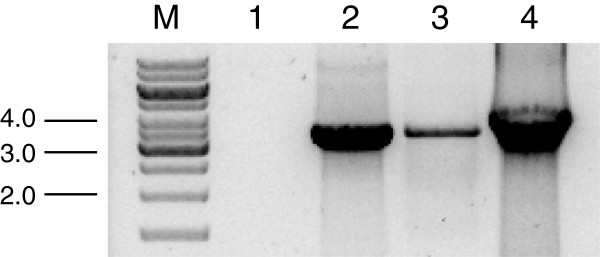
**Confirmation of integration of a full-length *tga3***^***Q207L ***^**gene in ectopic co-transformants 3/3 and 4/5.** The presence of a complete copy of the *tga3*^*Q207L*^ gene in co-transformants 3/3 (lane 2) and 4/5 (lane 3) was confirmed by PCR using primers tga3PromFw, which binds to the 5′ non-coding region of *tga3,* and M13Fw, which binds to the backbone of plasmid pKAtga3 (Table 1). Both co-transformants yielded the expected 3343-bp amplicon. Lane 1, wild-type; lane 4, plasmid pKAtga3.

Southern analysis was performed to confirm that the *tga3*^*Q207L*^ gene had been successfully integrated into the genome of the geneticin-resistant transformants. Two bands (1703-bp and 2272-bp) resulting from the endogenous *tga3* gene were observed in the wild-type after digestion of its genomic DNA with *Sac*I (Figure 5A and B). While transformant 3/3 exhibited two additional bands indicative for an ectopic single-copy integration of the *tga3*^*Q207L*^ gene, only one band in addition to those resulting from the endogenous *tga3* gene locus was observed in transformant 4/5 (Figure 5A and B). To discern whether two bands are overlying each other in the Southern blot or whether this banding pattern indicates integration of a truncated copy of *tga3*^*Q207L*^ in transformant 4/5, the presence of the full-length *tga3*^*Q207L*^ expression cassette was tested by PCR using primers tga3PromFw and M13Fw (Table 1). For both transformants a specific amplicon of 3343-bp was obtained (Figure 6), confirming integration of a complete *tga3*^Q207L^ expression cassette into the genome of transformant 4/5. To show that the integrated *tga3*^Q207L^ gene actually is expressed, *tga3* transcripts were sequenced. Four and two out of nine sequenced transcripts represented the mutated *tga3* allele in the ectopic co-transformants 3/3 and 4/5, respectively, proving expression of the *tga3*^Q207L^ gene copy.

To detect putative homologous recombinants among the remaining 20 transformants that showed the wild-type banding pattern in the Southern analysis, the *tga3* gene locus was amplified by PCR. To this end, a primer which anneals upstream of the 5^′^ flanking sequences present in the *tga3*^*Q207L*^ expression cassette (Figure 5A and Methods section) was used. The resulting amplicon was digested with *Bpu10*I. An additional *Bpu10*I recognition site is present in *tga3*^*Q207L*^ which originated from introducing the A to T mutation (Figure 5A). In 13 transformants *Bpu10*I digestion resulted in the 1004-bp and 1392-bp fragments indicative for the introduced mutation indicating that these are co-transformants with *tga3*^Q207L^ integrated at the homologous *tga3* gene locus (Figure 5C).

In summary, 15 out of 22 transformants (68%) with a stable integration of *nptII* had additionally integrated the *tga3*^*Q207L*^ gene into their genomes. A comparative co-transformation of *T. atroviride* with pKAtga3 (harboring *tga3*^Q207L^) and pAN7-1 (harboring the hygromycin B phosphotransferase-encoding *hph* gene) resulted in a co-transformation rate of 43% (data not shown). The yield of co-transformants obtained in this study by using either *nptII* or *hph* as selection marker is in accordance with previous publications reporting co-transformation efficiencies of 30 - 50% for *T. harzianum* and 90% for *T. longibrachiatum*[21-24].

### Phenotypic characterization of co-transformants

The Gα subunit Tga3 was previously shown to play crucial roles for vegetative growth and mycoparasitism-relevant signal transduction processes in *T. atroviride*. *Δtga3* mutants exhibited drastically reduced growth rates compared to the parental strain and were avirulent, i.e. unable to parasitize known susceptible host fungi [10].

**Figure 7:**
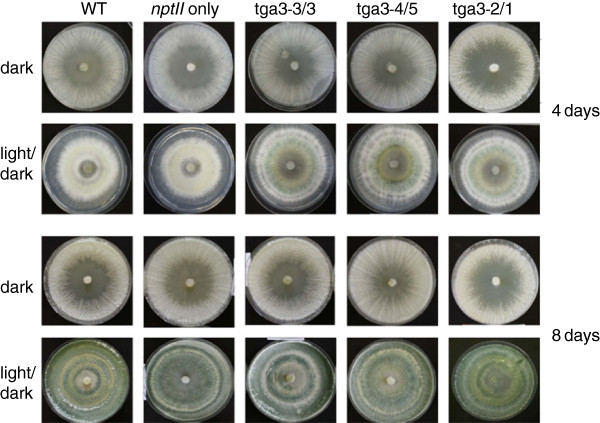
**Phenotype of *nptII/tga3***^***Q207L ***^**co-transformants.** Colony morphology of co-transformants 3/3 and 4/5 (with ectopic integration of the *tga3*^*Q207L*^ gene) and co-transformant 2/1 (with homologous integration of the *tga3*^*Q207L*^ gene) in comparison to the *T. atroviride* wild-type and a transformant having integrated *nptII* only upon growth on PDA for 8 days at 28°C either in the dark or under alternating light/dark (8/16 h) conditions. Co-transformants with either ectopic or homologous integration of *tga3*^*Q207L*^ showed normal light-induced conidiation behavior. Pictures were taken after 4 and 8 days.

For further analyses, co-transformants 3/3 and 4/5, which harbor the constitutively active Tga3 allele integrated ectopically, and co-transformant 2/1, which has integrated *tga3*^*Q207L*^ at the homologous locus, were used. All three co-transformants showed a ~20% increased growth rate compared to the wild-type and a transformant only harboring *nptII* (Table 2). While *Δtga3* mutants continuously produced spores also in the dark [10], *tga3*^*Q207L*^ co-transformants showed a conidiation behavior similar to the wild-type (Figure 7), with the characteristic feature being production of concentric rings of conidiophores in response to the light/dark cycle as previously described for T. atroviride *T. atroviride* e.g. [25]. Furthermore, the number of produced conidia was similar to that of the wild-type and the *nptII* transformants (Table 2).

**Figure 8:**
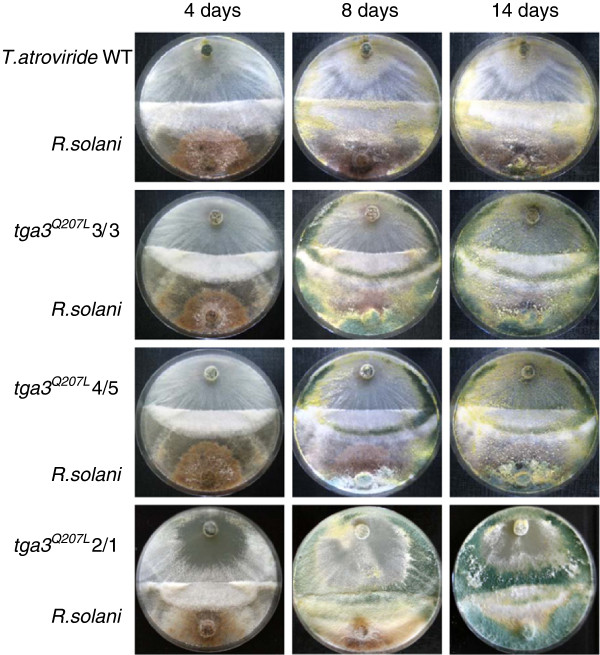
**Mycoparasitic abilities of co-transformants.** The antagonistic activity of co-transformants 3/3 and 4/5 (with ectopic integration of the *tga3*^*Q207L*^ gene) and co-transformant 2/1 (with homologous integration of the *tga3*^*Q207L*^ gene) in comparison to the *T. atroviride* wild-type was assessed in plate confrontation assays using *R. solani* as host fungus. While after four days of incubation at 28°C all strains showed a similar onset of mycoparasitism, after eight and 14 days the co-transformants exhibited more advanced overgrowth and host lysis compared to the wild-type.

Previous studies furthermore showed that *T. atroviride Δtga3* mutants were unable to attack and parasitize host fungi [10], while *T. reesei* mutants expressing the constitutively active Gα protein *gna3*QL exhibited a higher efficiency of antagonism against *Pythium ultimum*[26]. The effect of the constitutively active Tga3^Q207L^ protein on the mycoparasitic interaction between *T. atroviride* and the host fungus *R. solani* was assessed in plate confrontation assays. At the onset of mycoparasitism, *tga3*^*Q207L*^-expressing co-transformants behaved similar to the wild-type, whereas after eight and 14 days of incubation the co-transformants showed faster and more advanced mycoparasitic overgrowth and host lysis (Figure 8). These results reveal that constitutive signaling via Tga3 leads to enhanced mycoparasitism in *T. atroviride.*

## Conclusions

In this study we present the successful establishment of the neomycin phosphotransferase-encoding *nptII* gene from *E. coli* as a robust selection marker for the genetic transformation of the biocontrol fungus *T. atroviride*. Respective transformants showed stable geneticin resistance, had no unwanted phenotypic alterations, and their phenotypes were similar both in the presence and absence of geneticin (Additional file 1). The fact that no spontaneous geneticin-resistant *T. atroviride* colonies were detected makes the *nptII* selection marker a valuable alternative to the commonly used Hygromycin B phosphotransferase-encoding *hph* gene.

Co-transformation of *T. atroviride* with *nptII* and the *tga3*^*Q207L*^ gene on two different plasmids resulted in a high number of colonies with stable geneticin resistance. 15 out of 22 (68%) tested transformants were real co-transformants also having integrated *tga3*^*Q207L*^. Interestingly, the majority of the co-transformants showed an integration of *tga3*^*Q207L*^ at the homologous *tga3* gene locus. Despite the fact that both plasmids were used in their circular forms for transformation, this high number of homologous recombinants may have resulted from the long flanking regions present in the *tga3*^*Q207L*^ expression cassette which may have favored homologous double crossover events.

Constitutive activation of Tga3-mediated signaling led to an enhanced ability of *T. atroviride* to antagonize the phytopathogenic fungus *R. solani* thereby confirming the important role of G protein signaling in mycoparasitism.

## Methods

### Strains and media

*Trichoderma atroviride* strain P1 (ATCC 74058; teleomorph *Hypocrea atroviridis*) was used throughout this study. Fungal cultures were maintained on potato dextrose agar (PDA; Merck, Germany) at 28°C until intense sporulation. *Rhizoctonia solani* strain 1450 (strain collection of the Institute of Plant Pathology, University of Naples Federico II, Italy) was used as a plant pathogenic host. *Escherichia coli* JM109 served as a host for plasmid amplification and was grown as described by [27].

### Sensitivity of *T. atroviride* mycelium and conidia to geneticin (G418)

Plasmid pII99 [19] harbors the neomycin phosphotransferase-encoding *nptII* gene under control of the regulatory sequences of *A. nidulans trpC* (encoding a trifunctional protein involved in tryptophan biosynthesis, [28])*. nptII* confers resistance to geneticin (G418) and therefore allows the selection of transformed colonies on media containing this antibiotic. To determine the concentration of geneticin lethal for untransformed *T. atroviride* mycelia and conidia, either agar plugs covered with fungal mycelium or 10^3^ conidia were placed on increasing concentrations (0–100 μg/ml) of geneticin (Roth, Karlsruhe, Germany) on PDA. A geneticin concentration of 80 μg/ml not allowing any growth of *T. atroviride* was used for transformant selection.

### DNA procedures

Standard molecular techniques were performed according to [27]. Isolation of fungal DNA was carried out as described previously [29]. For standard PCR amplifications, recombinant *Taq* Polymerase (Fermentas, Vilnius, Lithuania) was used.

### Expression plasmid construction and fungal transformation

The Phusion Site-Directed Mutagenesis Kit (Finnzymes, Vantaa, Finland) was used to construct a mutated version of *tga3* [GenBank: AF452097], *tga3*^Q207L^, encoding a constitutively activated Gα protein with a single amino acid modification (Q207L). An equivalent mutation of the Tga3 orthologue in mammals resulted in the impairment of its intrinsic GTPase activity [30]. Corresponding mutations were introduced into other fungi in order to evaluate the function of Gα subunits e.g. [20,31,32]. Two oligonucleotides (tga3PhusionFw and tga3PhusionRev) (Table 1) were designed based on the *T. atroviride* genomic *tga3* sequence with tga3PhusionFw harboring the single-point mutation. In a PCR approach these oligonucleotides were used to amplify the *tga3* gene including approximately 1000-bp of 3^′^ and 5^′^ non-coding sequences in plasmid pGEM-T (Promega, Madison, WI). The linear amplified target plasmid with the mutated *tga3*^Q207L^ gene was re-circularized by ligation resulting in pKAtga3. The *tga3*^Q207L^-harboring insert was verified by sequencing to confirm that only the desired mutation had been inserted.

*T. atroviride* protoplasts were transformed with 10 μg of plasmid pII99 only or 10 μg of pKAtga3 in co-transformation with 2 μg of pII99 according to [33].

### Recovery of transformants and molecular analysis

Putative transformants were selected on PDA plates containing 80 μg/ml geneticin, a concentration which prevented the growth of *T. atroviride* strain P1. Transformants were transferred individually onto PDA supplemented with 80 μg/ml geneticin. Geneticin-resistant strains were individually transferred to PDA for sporulation and colonies obtained from three subsequent single spore isolation steps were used for further analysis. The presence of *nptII* was tested by PCR using oligonucleotides pII99Fw and pII99Rev (Table 1). For the purpose of comparison, hygromycin-resistant transformants were obtained by transforming *T. atroviride* with 10 μg of plasmid pAN7-1 [34] only or 10 μg of pKAtga3 in co-transformation with 2 μg of pAN7-1. Transformants were selected on 200 μg/ml hygromycinB (Calbiochem, San Diego, USA).

Integration of *tga3*^*Q207L*^ into the genome was analyzed by Southern blotting in transformants exhibiting a positive result in *nptII* screening. Standard molecular methods [27] were used for DNA electrophoresis and blotting. DNA labeling, hybridization and detection were performed according to the DIG High Primer DNA Labeling and Detection Starter Kit I protocols (Roche Applied Science). The DIG-labeled probe was amplified from pKAtga3 with oligonucleotides tga3ProbeFw and tga3ProbeRev (Table 1) producing an amplicon of 1118-bp. For detecting homologous recombinants among the transformants, the *tga3* gene locus was amplified by PCR using Phusion High-Fidelity DNA Polymerase (Thermo Fischer Scientific, Waltham, USA) and primers tga3-locus-check (which anneals upstream of the *tga3* 5^′^ flanking sequences present in the *tga3*^*Q207L*^ expression cassette) and tga3TermRev. The resulting amplicon was digested with *Bpu10*I and the banding pattern analyzed by agarose gel electrophoresis.

To prove that the *tga3*^*Q207L*^ gene copy was indeed expressed, co-transformants 3/3 and 4/5 were grown on PDA plates for 3 days, RNA isolated and reverse-transcribed to cDNA, and fragments of the *tga3* gene were amplified using primers Mut-tga3Fw and Mut-tga3Rev (Table 1). After cloning the resulting 604-bp fragments into pGEM-T (Promega, Madison, WI), the inserts of nine colonies representing *tga3* transcripts were sequenced to determine the ratio of wild-type and mutated alleles.

### Phenotype analysis of transformants

For assessing growth of *T. atroviride* transformants and wild-type, 5 mm diameter mycelial agar plugs of the respective strain were placed in the center of a PDA plate and incubated at 28°C. Radial growth was measured every 24 h until the plate was fully covered. Conidia were quantified by plating a freshly harvested and filtered spore suspension of the respective strain containing 1 × 10^6^ conidia onto PDA plates. Conidia produced on three independent plates after three days of incubation at 28°C were harvested and counted.

For plate confrontation assays, 5 mm mycelial agar plugs of the respective *T. atroviride* strains and *R. solani* as host were placed on PDA plates at a distance from each other of 4 cm and incubated in the dark at 28°C for 14 days. Pictures were taken at days four, eight, and 14.

## Abbreviations

BCA: Biocontrol agent; PDA: Potato dextrose agar; Bp: Base-pair; h: Hour.

## Competing interests

The authors declare that they have no competing interests.

## Authors’ contributions

SZ conceived and designed the study and wrote parts of the manuscript. SG and MO participated in the design of the experiments, generated the transformants and contributed to manuscript writing. SG, CER, and TR performed phenotypic analyses. All authors read and approved the final manuscript.

## Supplementary Material

Additional file 1 **Phenotype of *nptII / tga3***^***Q207L ***^**co-transformants on PDA and PDA + 80μg/ml geneticin.** The figure shows the colony morphology of co-transformants 3/3 and 4/5 (with ectopic integration of the *tga3*^*Q207L*^ gene) and co-transformant 2/1 (with homologous integration of the *tga3*^*Q207L*^ gene) grown on PDA and PDA + 80μg/ml geneticin for 4 days at 28°C in the dark. Click here for file
