# Supplementary figures and images for: Generation of Trichoderma atroviride mutants with constitutively activated G protein signaling by using geneticin resistance as selection marker
Source: BMC Res Notes. 2012 Nov 17;5:641. doi: 10.1186/1756-0500-5-641 (PMC3563614; doi:10.1186/1756-0500-5-641)

PDA

PDA + geneticin

tga3-3/3

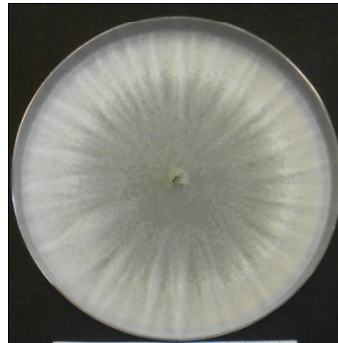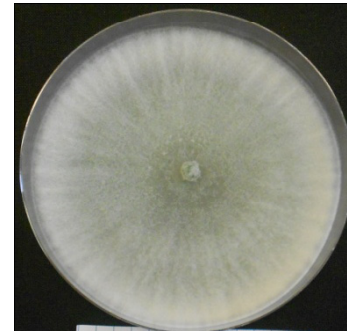

tga3-4/5

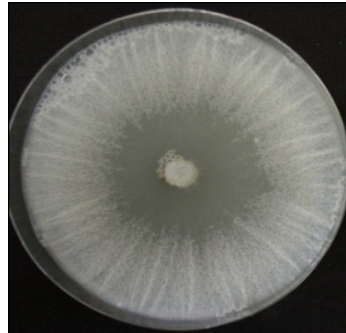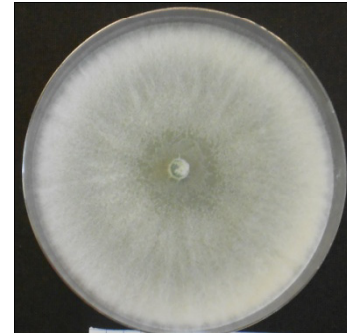

tga3-2/1

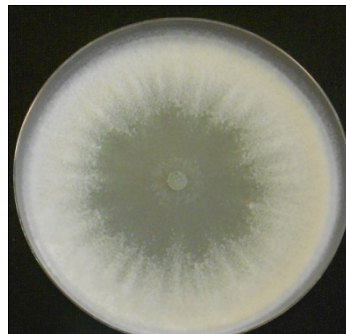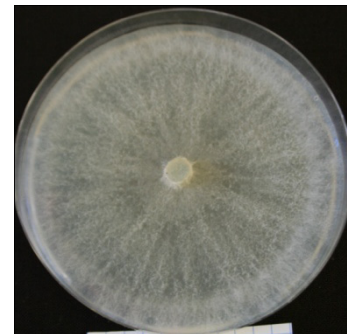

Supplement: Additional file 1 — Phenotype of nptII / tga3Q207L co-transformants on PDA and PDA + 80μg/ml geneticin. The figure shows the colony morphology of co-transformants 3/3 and 4/5 (with ectopic integration of the tga3Q207L gene) and co-transformant 2/1 (with homologous integration of the tga3Q207L gene) grown on PDA and PDA + 80μg/ml geneticin for 4 days at 28°C in the dark. [file 1756-0500-5-641-S1.pdf]
